# Breast Cancer Presenting As Onconeural Antibody Negative Opsoclonus-Myoclonus Syndrome

**DOI:** 10.7759/cureus.28417

**Published:** 2022-08-25

**Authors:** Ryan Soares, Amrutha Mittapalli, Manju Ramakrishnan, Umar Farooq

**Affiliations:** 1 Internal Medicine, St John's College, Philadelphia, USA; 2 Internal Medicine, Knights Medical Associates, Philadelphia, USA

**Keywords:** central vertigo, ataxia, paraneoplastic neurological syndromes, breast cancer, opsoclonus-myoclonus syndrome

## Abstract

Breast cancer can rarely present with or be preceded by paraneoplastic syndromes such as opsoclonus-myoclonus syndrome (OMS). OMS is a rare neurological syndrome that commonly presents with symptoms of rapid, chaotic eye movements (opsoclonus), jerking involuntary muscle movements (myoclonus) and is frequently associated with ataxia. We describe a case of a woman in her early 50s who presented to the emergency room (ER) with vertigo, jerking movements, loss of fine motor skills and gait abnormalities. She was initially thought to have likely vestibular neuritis and was treated symptomatically and discharged home. However, the symptoms persisted and she presented once again to the ER, at which time she also incidentally discovered a lump in her breast. This led to her being investigated more extensively leading to a diagnosis of underlying primary breast cancer. Based on her neurological clinical findings, she was diagnosed with onconeural antibody negative OMS. Treatment of her underlying malignancy led to a significant improvement in her symptoms. Paraneoplastic neurological syndromes (PNSs) are an important differential diagnosis to consider in patients presenting with persistent, treatment-resistant and non-specific neurological symptoms. Any suspicion of the same should prompt a search for an underlying malignancy that could greatly influence patient outcomes.

## Introduction

Opsoclonus-myoclonus syndrome (OMS) is a rare disorder of the nervous system that is characterized by arrhythmic, repetitive, multidirectional conjugate eye movements (opsoclonus), myoclonus, and ataxia. It is often accompanied by behavioral changes in adults and irritability in children. Sometimes, the myoclonus may precede the ophthalmic signs and can be aggravated by excitement or stress [1]. OMS could arise due to self-limiting parainfectious brainstem encephalitis or it may present as a paraneoplastic neurological syndrome (PNS) due to underlying cancers such as neuroblastomas in children, small cell carcinoma of the lung, and rarely breast adenocarcinomas in adults. Rarely, other etiologies such as metabolic and genetic syndromes are implicated. As the pathophysiology is largely believed to be immune-mediated, onconeural antibodies like anti-Hu and anti-Ri have been detected in the presence of a tumor and associated PNS, although a negative antibody test does not rule out a paraneoplastic etiology [2]. The diagnosis of PNSs is difficult due to a wide array of symptoms, timing of presentation, presence or absence of antibodies, and as was the case with our patient, manifestation prior to diagnosis of a primary tumor. In patients with underlying breast cancer, 80% of cases of paraneoplastic OMS are present prior to the diagnosis of malignancy [3]. We report this case in order to increase the understanding of the nature and timeline of symptoms that patients can present with, in order to have a high index of suspicion and reach a prompt diagnosis. We also discuss the current modalities of management of this particular syndrome cited in the literature.

## Case presentation

A 53-year-old female presented to the emergency room (ER) with complaints of severe vertigo, jerking movements of limbs, loss of fine motor skills and gait abnormalities, associated with nausea and vomiting for the past three days. The patient subjectively described the sensation as the room spinning around her. She also described a change in voice, photosensitivity, tinnitus, and fullness in her ears. She also described a sensation of her “eyes spinning out of control,” occurring several times a day along with the other symptoms. Prior to this episode, she was doing well with little significant medical history. She has a 26-pack-year smoking history and consumes alcohol occasionally. She attained menarche at age 14 and menopause at age 52. She had one vaginal delivery and used combined oral contraceptive pills for approximately six years. Her mother was diagnosed with metastatic lung cancer and metastatic melanoma in her early 70s, while her father was diagnosed with colon cancer in his late 70s.

**Figure 1:**
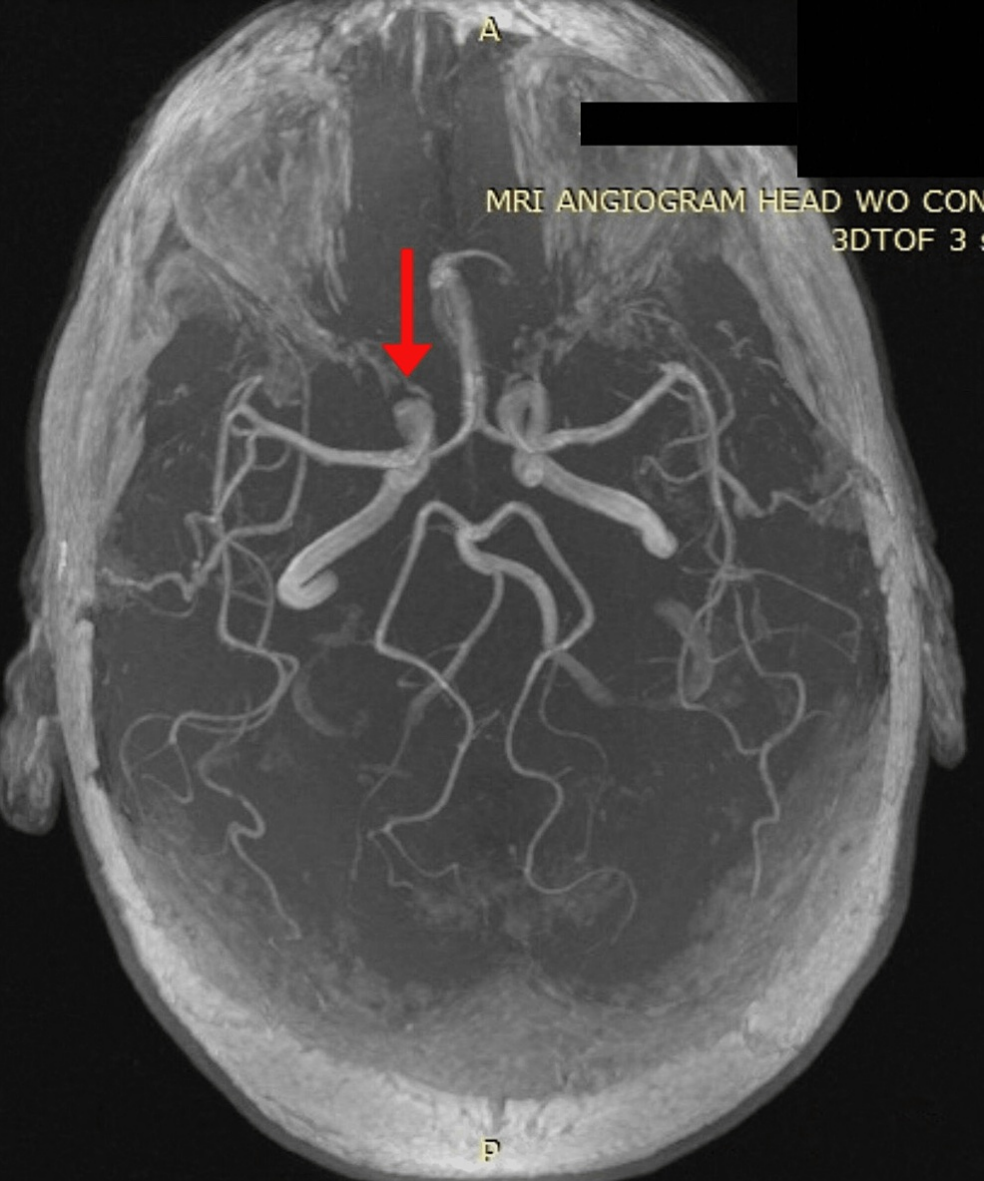
MR angiogram showing a 4 mm aneurysm in the right internal carotid artery (cavernous segment)

On admission, she was promptly investigated for a posterior circulation stroke with a computed tomography scan (CT scan) of the head and magnetic resonance angiography (MRA) which showed no evidence of a cerebrovascular accident (CVA) but showed an incidental 4 mm aneurysm in the right internal carotid artery in the cavernous segment (Figure 1). Due to her unremarkable findings, she was prescribed meclizine and discharged home with follow-up as necessary.

**Figure 2:**
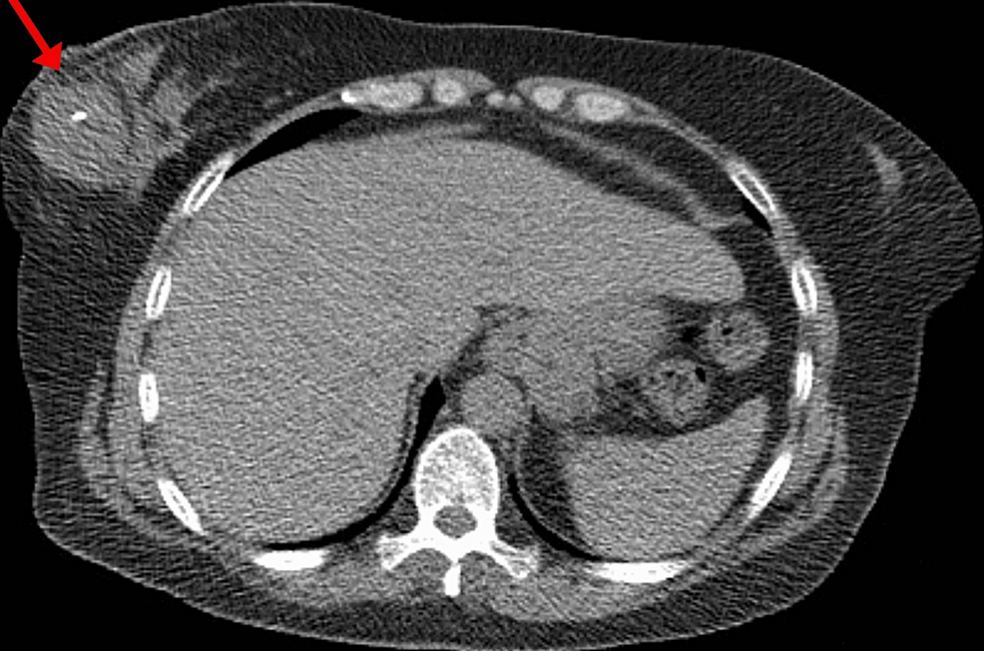
CT scan showing a 4.2-cm right breast mass (axial view)

**Figure 3:**
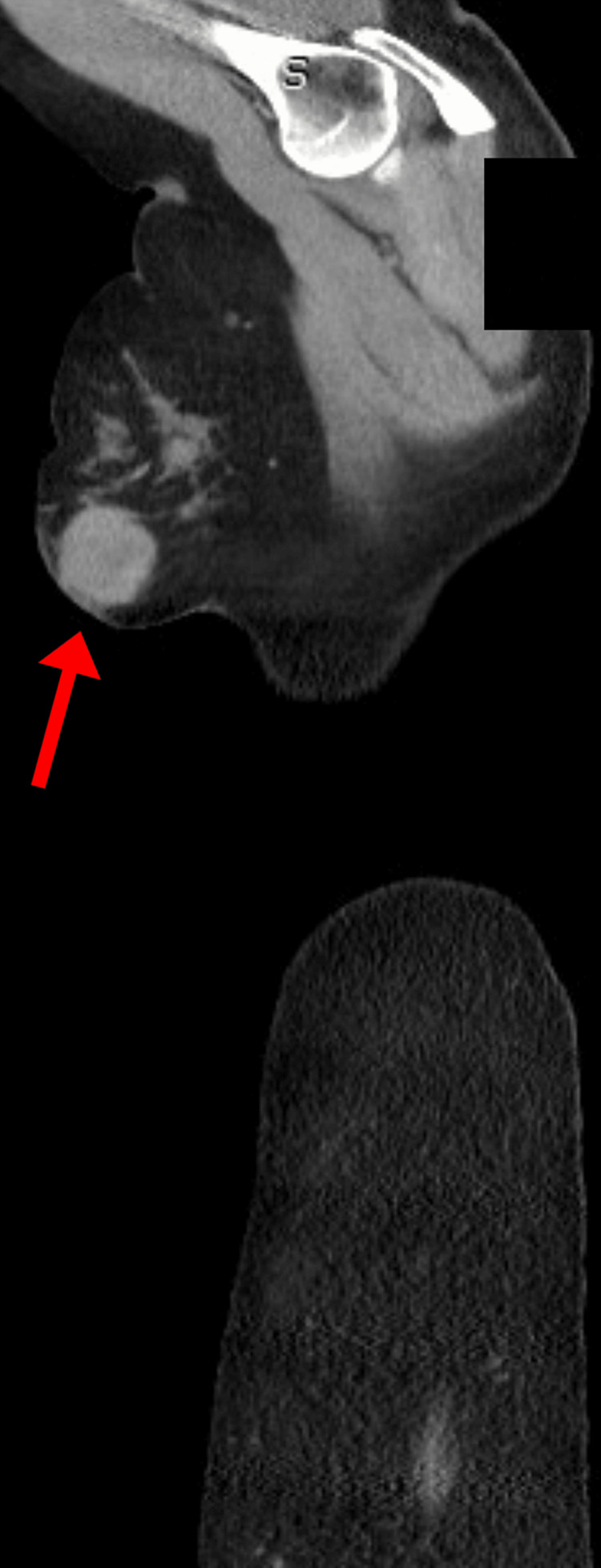
CT scan showing a 4.2-cm right breast mass (sagittal view)

The symptoms did not improve despite using the medication and she presented to the hospital once again after a week with similar complaints where she was admitted for further evaluation. During the evaluation, the patient mentioned that she had palpated a lump in her right breast at home and hence, a whole-body evaluation with a CT was done. The CT revealed a 4.2-cm right breast mass (Figures 2, 3).

**Figure 4:**
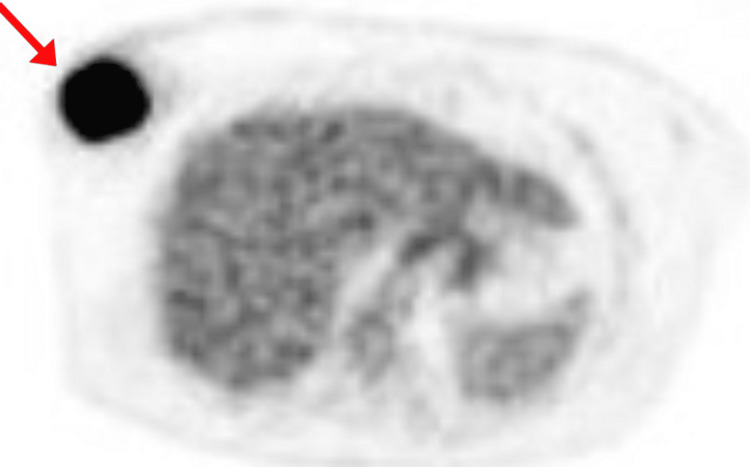
PET scan showing the primary tumor in the right breast

**Figure 5:**
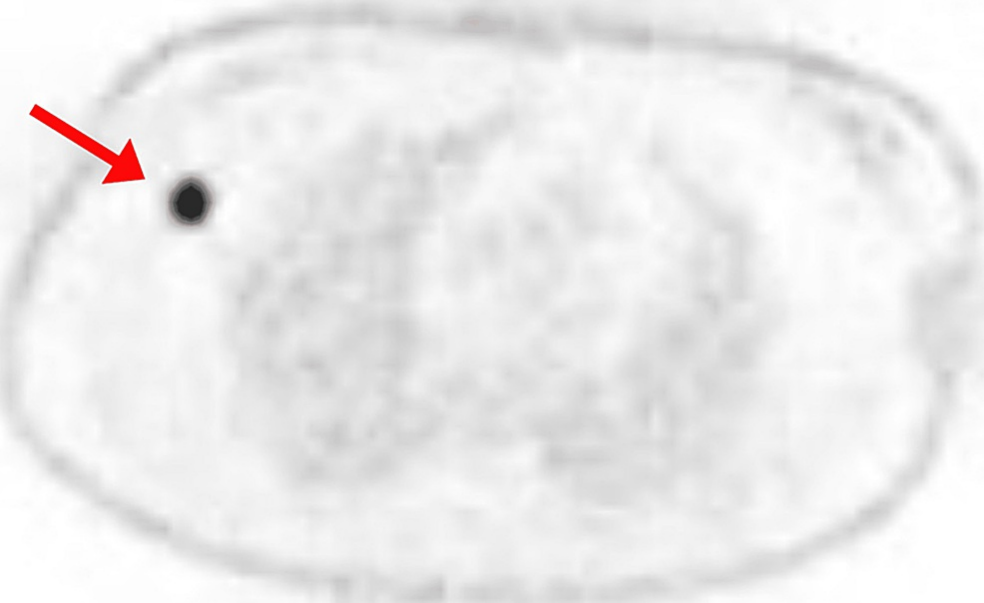
PET scan showing the 2.1 cm lymph node spread of the tumor

A PET/CT to localize the primary tumor and assess for possible distant metastases was ordered, which was then thought to be the cause of her presentation. In addition to the breast mass, a 2.1-cm right axillary lymph node was identified with no evidence of other distant metastases (Figures 4, 5). Subsequently, a 3D mammogram with tomosynthesis of the right breast was reported with a Breast Imaging-Reporting and Data System (BIRADS) score of six. The mammogram revealed three nodules as follows:

Nodule 1: lower outer quadrant (5.0 x 4.0 x 4.4 cm), 4 cm from the nipple.

Nodule 2: upper outer quadrant, (2.0 x 1.0 cm), 8 cm from the nipple.

Nodule 3: upper outer quadrant (in the axillary tail), (2.0 x 2.0 cm), 11 cm from the nipple.

A biopsy of all three nodules was taken, of which (Nodule 1) was malignant (invasive high-grade ductal carcinoma) and was found to be triple-negative for the estrogen and progesterone receptor (ER, PR) and HER-2/neu. She was diagnosed with stage IIB (T2N1M0 as per the TNM staging for breast cancer). A neurology consult was obtained for her ongoing vertigo and ataxia. Further investigation with a brain MRI showed no significant findings and a lumbar puncture was negative in terms of cytology, microbiology, and onconeural antibodies. Despite her negative findings, her clinical symptoms were highly suggestive of a PNS. Her case was discussed with a highly specialized multidisciplinary team and she was diagnosed as having onconeural antibody-negative paraneoplastic OMS.

She was initially treated with five doses of intravenous methylprednisolone resulting in minimal improvement in her symptoms. The team of experts decided to start her on neoadjuvant chemotherapy (adriamycin, cyclophosphamide, and paclitaxel) with rituximab to treat her PNS. The current standard of care also includes pembrolizumab but was not administered to this patient for fear of worsening the PNS. Intravenous immunoglobulins (IVIG) were thought to be of limited benefit in patients with ER/PR negative breast cancer. Neoadjuvant chemotherapy was administered over a period of six months and the tumor was downsized to 2 mm. She then underwent a wide local excision and a targeted axillary node dissection followed by adjuvant radiotherapy given over a duration of six weeks. Following tumor removal, her PNS symptoms started improving. At present, three months later, she only reports occasional episodes of vertigo and a fine hand tremor.

## Discussion

OMS, also known as “dancing eyes, dancing feet syndrome” is a heterogenous immune-mediated neurological manifestation. Opsoclonus consists of involuntary, arrhythmic, multidirectional, high amplitude and conjugate ophthalmic saccades. It is aggravated by pursuit fixation and persists upon eye closure and sleep. Distinguishing opsoclonus from other conditions is important, especially in arriving at a paraneoplastic etiological diagnosis. It can be distinguished from ocular flutter in that the latter has eye movements confined to the horizontal plane [4]. Opsoclonus complicates many neurological pathologies, but when it occurs as a PNS, it is often accompanied by myoclonus, truncal ataxia, and/or encephalopathy. PNS has distinct clinical features as compared to paraneoplastic cerebellar degeneration (PCD), of which opsoclonus was previously thought to be an extension [5].

The etiology of OMS may be idiopathic, paraneoplastic, postinfectious or metabolic in nature, and the most common cause of OMS is reported to be of paraneoplastic origin. The commonly associated neoplasms include small cell lung carcinoma and breast carcinoma [6,7]. Immune-mediated mechanisms involving both humoral and cell-mediated immunity are implicated in the course of the disorder, and a variety of paraneoplastic and cell-surface antibodies were found to be involved in many reported cases of paraneoplastic OMS. Ri/ANNA2 antibodies are good predictors of paraneoplastic OMS related to breast cancer while other antibodies are infrequently found [8]. OMS associated with anti-Ri antibodies cross-react with two antigens, Nova-1 and Nova-2 that are widely expressed within the CNS. Whilst many patients with breast cancer-associated OMS have antibodies present in their CSF, cases without antibodies do occur as observed in our case. This is likely explained by unknown antibodies or a paraneoplastic phenomenon mediated by activated T-cells [9].

The clinical features of OMS can vary based on the etiology. Individuals can additionally present with features like dysarthria, oscillopsia (illusory motion of the visual world), and vertigo as well as sleep disturbances, cognitive impairments, and behavioral changes. A retrospective cohort study evaluating 114 adult patients with OMS states that the symptoms of OMS preceded the diagnosis of the tumor with no differences among tumor types in the case of paraneoplastic OMS [10]. A clinical case series by L. Bataller also reported patients with paraneoplastic OMS were more likely to be older (>50 years), develop encephalitis, have a more aggressive disease course, and were likely to respond to immunotherapy when compared to idiopathic OMS [11]. The presentation of OMS also varies widely in terms of its chronology, onset, and neurologic course. In a study with 19 patients with paraneoplastic OMS, 73% of patients had early onset vertigo, nausea and vomiting and all but one patient had severe truncal ataxia. 63% of patients had encephalopathic features such as lethargy and confusion while one patient rapidly progressed to a comatose state [12].

Diagnosis of a paraneoplastic syndrome has been an enigma as it is often difficult to differentiate between a true PNS versus neurological syndromes co-existing with cancer. An international panel of neurologists convened to form a PNS-care panel in 2019 to review existing diagnostic criteria and developed the PNS-care scoring system [13]. The set of criteria taken into consideration include clinical phenotype, antibody type, presence/absence of cancer, and time length of follow-up. They put forward four levels of diagnostic certainties: “definite” (score>8), “probable” (scores 6-7), “possible” (scores 4-5) and non-PNS (scores ≤ 3). Due to the non-specific nature of symptoms, there is a broad range of differential diagnoses which include paroxysmal positional vertigo, vestibular neuritis, cerebellar strokes, encephalopathy, and brain tumors. It is also important to consider alcohol, amphetamines, phencyclidine, lithium, selective serotonin reuptake inhibitors (SSRIs), and other toxins as potential causes. Negative findings for investigating the above conditions should prompt further investigation for underlying malignancy with CT imaging and a PET scan should be considered if CT is inconclusive [14]. The presence of the anti-Ri onconeural antibody is highly suggestive of OMS and can aid in the diagnosis, but other reports have shown that this is variable. A report published by F. Ibrahim et al. described an anti-Ri positive patient with breast cancer presenting with ophthalmoplegia-ataxia syndrome with no myoclonic features [15].

In patients with OMS as a paraneoplastic phenomenon, treating the underlying malignancy is the most important component. The treatment usually includes surgery, chemotherapy, and/or radiotherapy. Additional management involves the use of immunotherapy, like IVIG, with or without corticosteroids and plasmapheresis. More intensive immunotherapy can be in the form of mycophenolate mofetil or rituximab. OMS as a manifestation of triple-negative breast cancer posed a therapeutic challenge. Guidelines for management must be individualized and integrated with the treatment of the primary tumor. Neoadjuvant chemotherapy and down-staging could help with paraneoplastic phenomena, but existing studies showed that symptoms could continue long after treatment of the primary tumor [16]. As was the case in our patient, breast cancer associated with paraneoplastic phenomena has been demonstrated to be more aggressive in its course [17]. Current treatment modalities include steroids, plasma exchange, IVIG, immunoadsorption, etc. Steroid therapy was of little benefit for our patient, once again highlighting the need for individualizing therapy. Although there are no set guidelines for the use of rituximab in OMS, it showed to be beneficial in this case. The use of rituximab is more commonly employed in children with refractory OMS, with no specific guidelines for its use in adults [18].

## Conclusions

OMS is a rare paraneoplastic syndrome that can rarely be associated with breast cancer. It is a difficult condition to diagnose due to its non-specific symptoms, varying chronological presentations, and possible overlapping features with other disorders. Additionally, as in this case, the classic diagnostic antibodies are not always identified. In breast cancer, it often presents before the diagnosis of the primary tumor and thus patients presenting with symptoms of opsoclonus, myoclonus and ataxia, which have no identified cause, should be thoroughly investigated for an underlying malignancy. Treatment regimens are highly variable and need to be individualized to each patient. Through this report, we hope to increase awareness and maintain a high clinical suspicion for this condition and also encourage the development of solid evidence-based treatment regimens for paraneoplastic OMS.
